# Left Ventricular Assist Device Infections and the Potential Role for Dalbavancin: A Case Report

**DOI:** 10.1093/ofid/ofz235

**Published:** 2019-09-04

**Authors:** Jessica Howard-Anderson, Stephanie M Pouch, Mary Elizabeth Sexton, Aneesh K Mehta, Andrew L Smith, George M Lyon, Rachel Friedman-Moraco

**Affiliations:** 1 Division of Infectious Diseases, Atlanta, Georgia; 2 Division of Cardiology, Department of Medicine, Emory University School of Medicine, Atlanta, Georgia

**Keywords:** dalbavancin, left ventricular assist devices, *Staphylococcus aureus*

## Abstract

Left ventricular assist device infections (LVADIs) are common but challenging to treat, often requiring prolonged courses of intravenous antibiotics. Dalbavancin could have a role in treating patients with chronic LVADIs given its less frequent dosing requirements. Here, we illustrate a case in which dalbavancin was used as suppressive therapy for an LVADI for greater than 7 months.

## CASE REPORT

**Figure 1.**
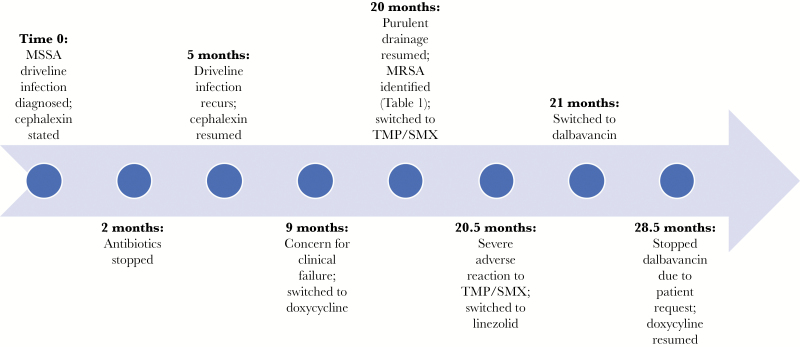
Timeline of Significant Events. MRSA indicates methicillin-resistant *Staphylococcus aureus*; MSSA, methicillin-susceptible *Staphylococcus aureus;* TMP/SMX, trimethoprim/sulfamethoxazole.

A 59-year-old female with diabetes, ischemic cardiomyopathy, and a left ventricular assist device (LVAD) placed 4 years prior as destination therapy had a history of recurrent LVAD infections (LVADIs). She developed a methicillin-susceptible *Staphylococcus aureus* (MSSA) driveline infection 2 years after LVAD placement and was treated with 2 months of cephalexin. The infection recurred after stopping antibiotics, and cephalexin was restarted. Four months later, she was switched to doxycycline out of concern for clinical failure, although her cultures persistently grew MSSA. She did well for 1 year until purulent driveline drainage recurred. Cultures grew MSSA and a new *S. aureus* strain, which was resistant to oxacillin, doxycycline, and clindamycin (Table 1). Blood cultures were negative. Therapy was switched to trimethoprim/sulfamethoxazole, but she developed a severe reaction, including kidney injury, hepatitis, and fevers, and was converted to linezolid. Given concern for toxicities with long-term linezolid, we switched her to dalbavancin 1500 mg intravenously (IV) weekly for suppressive therapy (Figure 1). After 10 weeks of therapy, we reduced the dose to 1500 mg every 2 weeks. She had 1 readmission 3 months into treatment for hyperglycemia due to diabetes and possible driveline exit site infection. Wound cultures grew *Klebsiella pneumoniae* (pan-susceptible) and *Serratia marcescens* (resistant to cefazolin and cefoxitin), as well as MSSA in 1 of 2 cultures, which was thought to represent skin colonization. She took levofloxacin for 2 weeks and continued dalbavancin, which she tolerated well with no adverse events and stable renal and hepatic function. After receiving dalbavancin for a total of 235 days, she requested a switch to oral therapy; given no further cultures identifying the prior resistant *S. aureus* strain, we resumed doxycycline.

**Table 1.** Antimicrobial Susceptibility Testing for the Final Methicillin-resistant *Staphylococcus aureus* Isolated From LVAD Driveline Purulent Drainage

	MIC (µg/mL)	Interpretation*
Clindamycin	0.25	Resistant^**^
Erythromycin	4	Resistant
Gentamicin	≤ 0.5	Sensitive
Inducible clindamycin resistance	Positive	Resistant
Oxacillin	1	Resistant^#^
Rifampin	≤ 0.5	Sensitive
Tetracycline	≥ 16	Resistant
Trimethoprim/sulfamethoxazole	≤ 10	Sensitive
Vancomycin	1	Sensitive

*Based off VITEK 2 (bioMerieux, Durham, NC) (card GP78) antibiotic susceptibility testing.

**Labelled as resistant because the inducible clindamycin resistance test was positive.

#Labelled as resistant because the isolate tested positive for penicillin-binding protein 2a, suggesting methicillin resistance.

MIC indicates minimum inhibitory concentration.

## DISCUSSION

Here, we illustrate a novel role for dalbavancin in treating LVADIs. Approximately one-third of patients develop an LVADI within 2 years of LVAD implantation, most commonly caused by gram-positive bacteria. LVAD exchange is associated with significant morbidity, making surgical management rare. Therefore, a majority of patients require suppressive antimicrobials after initial therapy [1, 2]. IV antibiotics can require multiple daily doses through vascular access devices, necessitating laboratory monitoring and home services. Given these challenges, treatment regimens that require less frequent administration, minimize vascular access devices, and prevent invasive procedures are appealing.

Dalbavancin is a second-generation lipoglycopeptide antibiotic with activity against many gram-positive organisms, including staphylococci, streptococci, and enterococci [3]. It is FDA-approved for acute bacterial skin and skin structure infections, although original trials excluded patients with device infections [4, 5]. Dalbavancin has an excellent safety profile, having only 3 treatment-related serious adverse events in a pooled analysis of almost 1800 patients [6].

Dalbavancin increasingly is being used for extended durations for deep-seated infections, having success reported in osteomyelitis [7–9], prosthetic joint infections, and endovascular infections, with the longest recorded treatment of 168 days [9]. Another study highlighted using dalbavancin to treat endocarditis; although, one patient developed resistance to dalbavancin after 210 days [10].

We are not aware of any studies investigating the use of dalbavancin for LVADI treatment or dosing for chronic therapy. The terminal half-life of dalbavancin is just over 2 weeks, and a single 1000 mg dose can provide plasma concentrations higher than the MIC_90_ for *S. aureus* for at least 14 days [11]. Dosing recommendations for patients on long-term therapy are not standardized, ranging from 500–1500 mg IV every 1 to 2 weeks [7, 9, 10]. The long half-life of dalbavancin also raises concerns for developing resistance, especially after cessation of therapy, with the potential for a prolonged mutant selection window [12]. We are only aware of 2 cases describing dalbavancin resistance during, or immediately following, therapy [13, 14]. One case involved treatment with 210 days of dalbavancin for a cardiac device infection before the isolate became dalbavancin nonsusceptible; it remained vancomycin susceptible [13]. The second patient was treated with 8 days of vancomycin and 1 dose of dalbavancin for a central venous catheter infection. Two weeks later, a urine culture grew *S. aureus* resistant to dalbavancin (MIC 0.5 µg/mL) and intermediate to vancomycin (MIC 4 µg/mL) [14]. The cost of dalbavancin also can be concerning (as the estimated wholesale price at our institution is $1700 for 500 mg dalbavancin); however, depending on the clinical scenario and alternative treatment options, use may be cost-effective [8, 15].

## CONCLUSION

This is the first case to describe dalbavancin as long-term therapy for an LVADI. Our patient did not have bacteremia, so this case may not be applicable to all patients with LVADIs. However, dalbavancin’s unique weekly dosing schedule and favorable safety profile make it a potentially valuable option for patients who require suppressive therapy for an LVADI with limited oral therapeutic alternatives.
